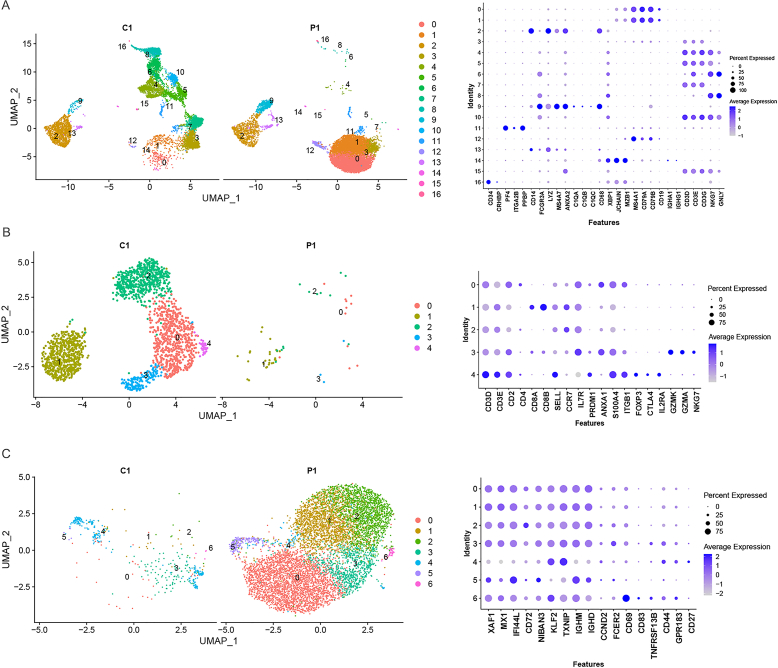# Identification of a splice site mutation in IL2RG in a Chinese boy with X-linked severe combined immunodeficiency

**DOI:** 10.1016/j.gendis.2025.101515

**Published:** 2025-01-04

**Authors:** Feng Ding, He Zhang, Xiangxiang Liu, Lin Lei, Hongyi Zhang, Zhichao Liu, Mutong Fang, Shuihua Lu

**Affiliations:** a National Clinical Research Center for Infectious Diseases, Shenzhen Third People’s Hospital, Shenzhen, Guangdong 518112, China; b Shenzhen Third People’s Hospital, The Second Affiliated Hospital, Southern University of Science and Technology, Shenzhen, Guangdong 518112, China

Interleukin 2 receptor gamma (*IL2RG*) is an important receptor component for interleukin-2 (IL2) family cytokines including IL2, IL4, IL7, IL9, IL15, and IL21.^1^ *IL2RG* is located on the X-chromosome q13.1, encoding a common gamma chain (γC) that is essential in lymphoid development, especially in the modulation of T cell and natural killer (NK) cell immune responses. Mutations in the *IL2RG* gene cause X-linked severe combined immunodeficiency (X-SCID), which is a life-threatening rare disease. In typical X-SCID, the disease is characterized by a nearly complete absence of T cells and NK cells, alongside normal or elevated counts of non-functional B cells (T^–^ B^+^ NK^−^ phenotype). Infants with X-SCID exhibit high susceptibility to bacterial and opportunistic infections. Here, we report a splice site mutation (c.924+5G > C) in the *IL2RG* gene in a 3-month-old boy presenting with a typical phenotype of X-SCID.

The proband was admitted to hospital due to recurrent fever at 3 months of age. He received Bacillus Calmette-Guérin (BCG) vaccination shortly after birth, subsequently developing disseminated BCG disease. *Mycobacterium tuberculosis* was detected in multiple specimens, including blood, respiratory samples, feces, and gastric juice. A pus spot was found on the BCG scar through physical examination. Furthermore, the proband was co-infected with *Nocardia farcinica*, detected in both blood and phlegm. The proband has a healthy elder brother. His mother experienced a miscarriage at 8 weeks, as well as another stillborn at 6 months, both confirmed to be male fetuses. There is no history of tuberculosis or genetic diseases among the family members. Investigations revealed severe T-cell deficiency: CD4^+^ T cells, 0.022 × 10^3^/uL (normal range: 0.6–1.1 × 10^3^/uL); CD8^+^ T cells, 0.003 × 10^3^/uL (normal range: 0.5–1 × 10^3^/uL) (Table S1). Immunoglobulin A and M levels were 1.6 mg/dL (normal range: 14–138 mg/dL) and 6.2 mg/dL (normal range: 38–144 mg/dL), respectively, indicating dysfunction of B cells. Chest computed tomography scan showed large consolidation with cavity formation in the left lower lobe of the lung, multiple patchy shadows in both lungs, slight pleura thickening on the right lung, and underdeveloped thymus (Fig. 1A). The proband was initiated on a regimen of anti-tuberculosis treatment comprising isoniazid, rifampicin, and ethambutol. Additionally, ceftriaxone sodium was administered as prophylaxis against bacterial infection. Due to the emergence of adverse reactions and persistent fever, the regimen was modified to include isoniazid, ethambutol, levofloxacin, and linezolid, along with linezolid and sulfamethoxazole for nocardiosis and meropenem for bronchopneumonia. Within 48 h of initiating this adjusted regimen, the proband's temperature markedly decreased and subsequently stabilized within the normal range.

**Figure 1:**
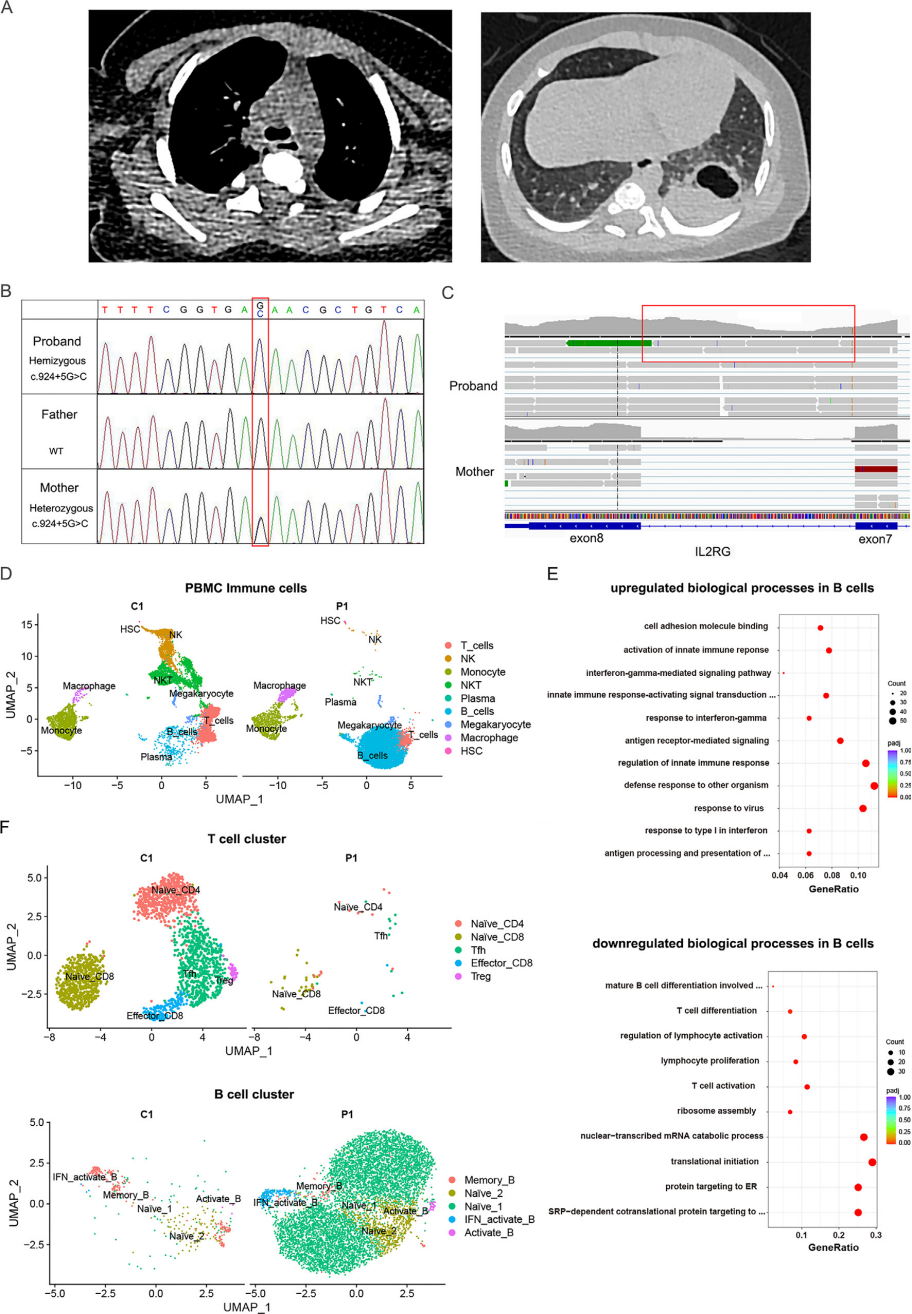
A X-SCID patient with hemizygous splice site mutation in *IL2RG*. **(A)** Chest computed tomography scan of the proband. **(B)** Genomic sequence of *IL2RG* gene in the proband carrying the hemizygous splice site mutation and his parents. The red box indicates the position where the nucleotide variant is located. **(C)** Schemes of novel *IL2RG* mRNA transcripts showing the intron retention identified by RNA sequencing in the proband (upper panel), compared with the normal mRNA transcripts identified in his mother (bottom panel). The red box indicates the identified intron retention. **(D)** UMAP plot of single-cell RNA sequencing data of peripheral blood mononuclear cells from the proband (P1) and the mother (C1). NK, natural killer; NKT, natural killer T; HSC, hematopoietic stem cells. **(E)** Gene ontology (biological processes) enrichment analysis based on differentially expressed genes (DEGs) of B cells. The size of the dots represents the number of genes in the significant DEG gene list associated with the gene ontology term and the color of the dots represents the *P*-adjusted values. **(F)** UMAP plot of T cell and B cell subpopulations in the peripheral blood mononuclear cells in (D). Tfh, T follicular helper cell; Treg, regulatory T cell; IFN, interferon.

Whole exome sequencing was performed to identify the causative genetic defects (supplementary methods). Whole exome sequencing detected a hemizygous splice site mutation (c.924+5G > C) in the *IL2RG* gene. This mutation, a G to C substitution at the fifth base of intron 7, was predicted to be deleterious using SpliceAI and MaxEntScan. The mutation was confirmed to be inherited from his mother and absent in his father by Sanger sequencing (Fig. 1B). The splice site mutation was not found in clinvar, gnomAD, Kaviar, dbSNP, or mBiobank, but it was previously reported in HGMD based on a study published in 1997.^2^ In that study, c.924+5G > C was detected in a patient who had a classic, severe infantile SCID phenotype. However, since neither *IL2RG* mRNA expression nor surface γc protein expression was evaluated in that study, further investigation is required to classify this splice site mutation as a pathogenic variant.

To investigate the consequences at the molecular level of this mutation, we performed RNA sequencing on blood samples from both the proband and his mother. RNA sequencing analysis confirmed that the splice site mutation resulted in aberrant mRNA splicing, generating a novel transcript with the retention of intron 7 (Fig. 1C). The intron retention caused the incorporation of five aberrant amino acid residues and a premature stop codon (Fig. S1), thus producing a truncated γC chain with 313 amino acid residues, shorter than the 369 amino acid residues encoded by the wild-type allele. Gene ontology enrichment analysis of differentially expressed genes revealed up-regulation of genes associated with defense responses and down-regulation of genes related to T cell activation and differentiation (Fig. S2A). Single-cell RNA sequencing was performed on peripheral blood mononuclear cells obtained from both the proband and his mother. The proband exhibited a significant reduction in T cells, with NK and NKT cells nearly absent. In contrast, the numbers of B cells and macrophages increased, while monocytes remained normal (Fig. 1D; Fig. S3A). We extracted the cell subpopulations and performed functional annotation (Fig. 1E; Fig. S2B–E). In B cells, genes related to defense responses were up-regulated, while genes associated with protein localization and translation, T cell activation and differentiation, and mature B cell differentiation were down-regulated, aligning with the results from RNA sequencing. Among the genes down-regulated in B cells, three (*LGALS1*, *GPR183*, and *DOCK10*) were known to be involved in mature B cell differentiation. The down-regulation of these genes may suggest an impaired transition of activated B cells towards plasma cells or memory B cells. Further analysis of T cell and B cell subclusters (Fig. 1F; Fig. S3B, C) revealed a significant reduction in all T cell subclusters in the proband, along with a notable increase in naive B cells and a decrease in memory B cells. These findings indicated that, while B cell proliferation appeared unaffected in the proband, the ability of naive B cells to differentiate into mature antibody-secreting cells was compromised, likely resulting in an inadequate antibody response to bacterial or viral invasions. Taken together, we concluded that the c.924+5G>C mutation, which led to a truncated γC chain, was a loss-of-function mutation. This mutation disrupted the development and differentiation of immune cells, particularly B cells, thereby contributing to the observed immune deficiencies in the proband.

Mutations in the *IL2RG* gene associated with X-SCID were first reported in 1993,^3^ and since then, more than 260 different pathogenic/likely pathogenic mutations have been identified throughout the *IL2RG* gene. Most of the mutations are frameshift, missense, or nonsense mutations. Additionally, 18 splice site defects have been reported as pathogenic or likely pathogenic in ClinVar (Table S2). Among them, eight were changes in the invariant gt found at the +1 and + 2 positions of 5′ splice sites, and one is a 2bp TG deletion. Nine 3′ splice site mutations were found at the −2 and −1 invariant ag. Besides, other splice site mutations were reported in published studies. For instance, although only c.924+1G > A was reported in ClinVar, other mutations at the same splice site have been observed in several patients with typical X-SCID, including c.924+1G > T, c.924+2T > G, and c.924+5G > C.^4^ Northern blot and cell surface immunofluorescence analysis revealed trace amounts of *IL2RG* mRNA and γC expression in patients with variations 924+1G > T and 924+1G > A. However, probably due to the lack of B-cell lines from patients, mRNA and γC expression detection were not performed for the other two variations (c.924+2T > G and c.924+5G > C). In our study, we identified that c.924+5G > C led to aberrant splicing of *IL2RG* mRNA. We also found that the mutation affected the development and differentiation of immune cells.

RNA sequencing offers the advantages of detecting RNA expression abnormalities and uncovering novel splice site variants.^5^ This present study applied single-cell RNA sequencing and bulk RNA sequencing to investigate the transcriptome landscape of an X-SCID patient. To our knowledge, this is the first report to identify c.924+5G > C as a loss-of-function inactivating mutation. Our findings expand the genetic etiology spectrum of X-SCID and provide novel insights into the mechanism underlying the *IL2RG*-mediated immune response.

## Ethics declaration

This study was reviewed and approved by the Ethics Committee of the Third People's Hospital of Shenzhen, Guangdong, China (approval number: 2022-198-02). The patient's data is confidential, used for research purposes only, and in compliance with the Declaration of Helsinki. Written informed consent for participation in this study was provided by the participant's legal guardians/next of kin.

## Funding

This work was supported by Shenzhen Science and Technology Program (Guangdong, China) (No. LCYX20220620105200001), Guangdong Basic and Applied Basic Research Foundation (Guangdong, China) (No. 2024A1515011959), Shenzhen Medical Research Fund (Guangdong, China) (No. C2405002), Shenzhen Fund for Guangdong Provincial High-level Clinical Key Specialties (Guangdong, China) (No. SZGSP010), and Shenzhen High-level Hospital Construction Fund (Guangdong, China) (No. G2022097).

## CRediT authorship contribution statement

**Feng Ding:** Conceptualization, Data curation, Formal analysis, Investigation, Methodology, Project administration, Software, Validation, Visualization, Writing – original draft, Writing – review & editing. **He Zhang:** Methodology. **Xiangxiang Liu:** Data curation, Investigation. **Lin Lei:** Data curation, Investigation. **Hongyi Zhang:** Data curation, Investigation. **Zhichao Liu:** Data curation, Investigation. **Mutong Fang:** Investigation, Project administration, Supervision, Writing – review & editing. **Shuihua Lu:** Conceptualization, Funding acquisition, Project administration, Supervision, Writing – review & editing.

## Conflict of interests

The authors declared no conflict of interests.

## Figures and Tables

**figs1:**
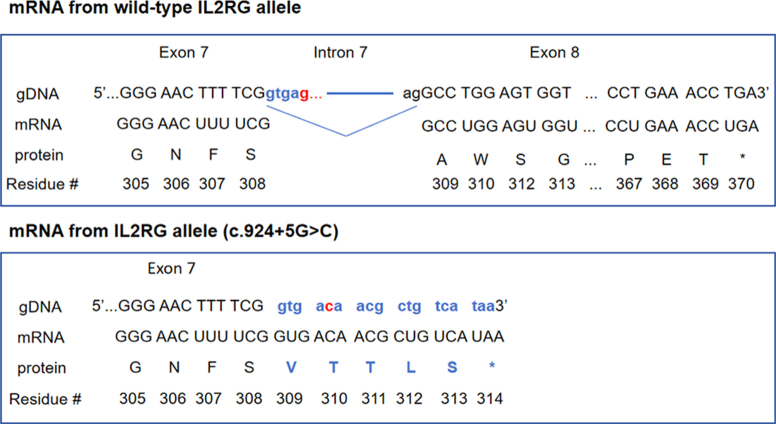


**figs2:**
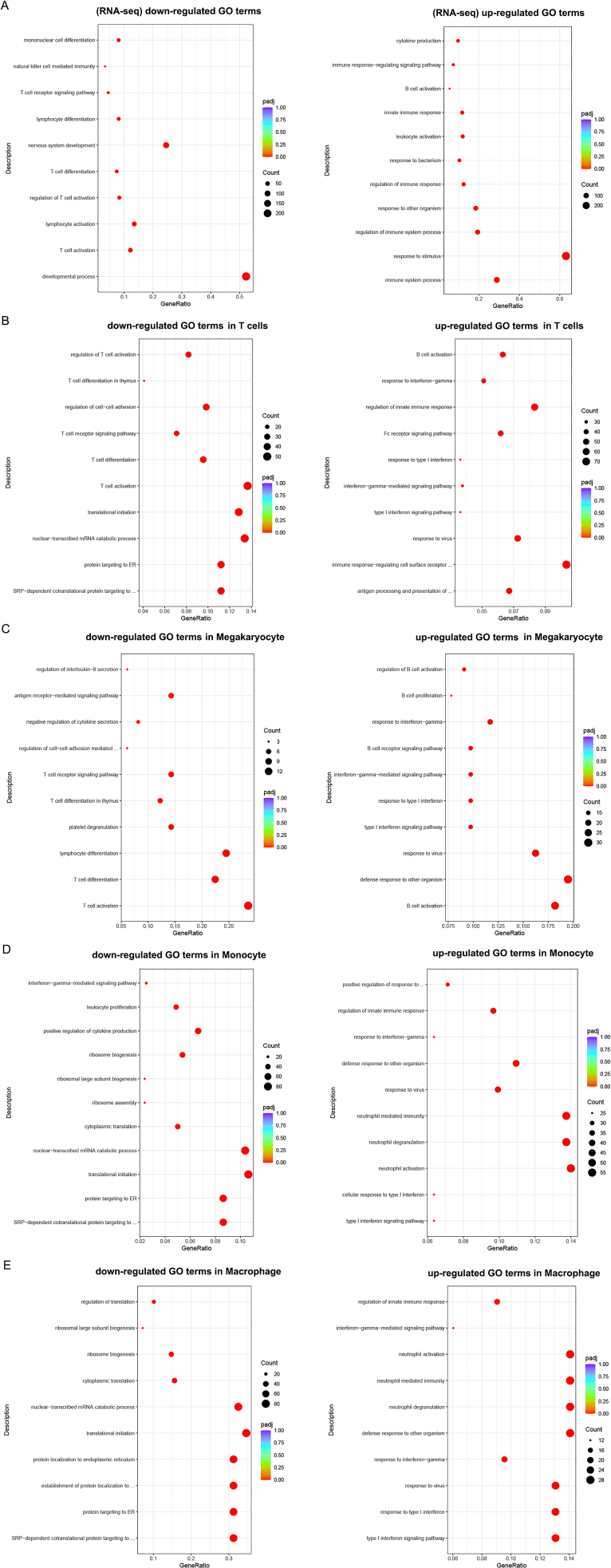


**figs3:**