# Supplementary material for: Identification of a splice site mutation in IL2RG in a Chinese boy with X-linked severe combined immunodeficiency
Source: Genes Dis. 2025 Jan 4;12(5):101515. doi: 10.1016/j.gendis.2025.101515 (PMC12148565; doi:10.1016/j.gendis.2025.101515)
Supplement: Multimedia component 1 [file mmc1.docx]

**Supplemental figures**

**Figure S1** mRNA transcript generated by the hemizygous splice site mutation (c.924+5G>C) in *IL2RG*. Overview of DNA, mRNA, and protein sequences of wild-type *IL2RG* allele (upper panels) and *IL2RG* allele containing the splice site mutation c.924+5G>C (bottom panels). In the DNA and mRNA sequences, capital letters indicate nucleotides from exons, lowercase letters indicate nucleotides from introns, and blue lines indicate normal mRNA splicing.

**Figure S2** Gene ontology (biological processes) enrichment analysis based on differentially expressed genes (DEGs) of bulk RNA sequencing data and single-cell RNA sequencing data. **(A)** Enrichment analysis based on significantly down-regulated and up-regulated DEGs of bulk RNA sequencing data. **(B)** Enrichment analysis based on significantly down-regulated and up-regulated DEGs of T cells. **(C)** Enrichment analysis based on significantly down-regulated and up-regulated DEGs of megakaryocytes. **(D)** Enrichment analysis based on significantly down-regulated and up-regulated DEGs of monocytes. **(E)** Enrichment analysis based on significantly down-regulated and up-regulated DEGs of macrophages. The size of the dots represents the number of genes in the significant DEG gene list associated with the gene ontology term and the color of the dots represents the *P*-adjusted values.

**Figure S3** Single-cell transcriptome landscape of the proband and the mother. **(A)** UMAP plot of single-cell RNA sequencing data of peripheral blood mononuclear cells from the proband (P1) and the mother (C1) (left) and expression of top markers for each cell cluster (right). **(B)** UMAP plot of subset analysis of T cells (left) and expression of top markers for each cell cluster (right). **(C)** UMAP plot of subset analysis of B cells (left) and expression of top markers for each cell cluster (right). As indicated in the legend, the dot size denotes the percentage of cells in a cluster expressing the gene. The dot color represents the relative average expression level.

**Materials and methods**

**Collection of clinical samples**

Peripheral blood from the proband and his parents were collected with written informed consent, according to procedures approved by the Shenzhen Third People’s Hospital (Approval number: 2022-198-02).

**Whole exome sequencing analysis**

Genomic DNA of the proband and his parents were extracted using the QIAamp DNA Blood Mini Kit (Qiagen, Germany), according to the manufacturer’s instructions. A total of 200 ng of genomic DNA of each individual was used for whole-exome sequencing (WES). Raw reads were filtered using FastQC and mapped to the human reference genome (GRCh37) by BWA[1]. Single nucleotide variants (SNVs) and insertions/deletions (indels) were detected following GATK best practice guidelines[2]. Variant filtering was performed based on inheritance patterns, functional annotation, phenotype correlations, and allele frequencies in reference databases including HGMD, Clinvar, OMIM, 1000Genome, dbSNP, ExAC, and GnomAD. Sanger sequencing was performed to validate the IL2RG mutation in the individuals. SpliceAI and SIFT was employed to predict whether the mutation affects mRNA splicing. The splice site mutation (c.924+5G>C) in IL2RG was verified in the read alignments (BAM files) using the Integrative Genomics Viewer (IGV, https://igv.org/).

**RNA-seq and data analysis**

Blood sample were collected from the proband and his mother in the PAXgene blood RNA tube (BD Biosciences). RNA was isolated using the QIAamp PAXgene Blood RNA Kit. 500g RNA was used for library preparation using Optimal Dual-mode mRNA Library Prep Kit (BGI-Shenzhen, China). Globin mRNA was removed by using Globin & rRNA Depletion Kit (Vazyme-Nanjing, China). First-strand cDNA was generated using random hexamer-primed reverse transcription, followed by a second-strand cDNA synthesis. The synthesized double strand cDNA was subject to end repairment reaction. After cDNA end repairment, a single ‘A’ nucleotide is added to the 3’ ends of the blunt fragments through a tailing reaction. Then the adaptors are connected to the double stranded cDNA with adaptor ligation reaction. Finally, the library products are amplified through PCR reaction and subjected to quality control. Next, cDNA libraries were sequenced on a DNBSEQ-T7 platform (BGI-Shenzhen, China) using paired-end 150-bp (PE150) reads.

Raw data was filtered with SOAPnuke (v1.5.2)[3]. Clean data was mapped to the human reference genome (hg38) by HISAT (v2.1.0)[4] and Bowtie2 software [5]. After mapping, featureCounts was used to count the reads numbers mapped to each gene[6]. Differential expression analysis was performed using the DESeq2(v1.34.0)[7]. According to the Gene Ontology (GO) and Kyoto Encyclopedia of Genes and Genomes (KEGG) annotation results and classifications, the differentially expressed genes were functionally classified, the phyper in R software was used for KEGG enrichment analysis, and the TermFinder package was used for GO Enrichment. With a Q value of ≤ 0.05 as the threshold, candidate genes that met this condition were defined as significantly enriched.

**Single-cell RNA-seq and data analysis**

**10× library preparation and sequencing**

Peripheral blood mononuclear cells (PBMCs) from the proband and the mother were isolated via Ficoll-Hypaque density gradient centrifugation (GE Healthcare, Boston, MA, U.S.A.). According to manufacturer’s instructions for the 10X Genomics Single Cell 3’ V3.1 Reagent Kit, prepared single-cell suspension were processed using the 10X Genomic Chromium Controller for partitioning and barcoding, after which cDNA libraries were generated. Sequencing was performed on MGI-2000 (BGI-Shenzhen, China) using paired-end 100-bp (PE100) reads.

**Data Preprocessing, Clustering and Differential Expression**

FASTQ files were aligned to the GRCh38 reference genome by running Cell Ranger pipelines (version 7.1.0) on 10X Genomics Cloud analysis[8]. The resulting gene-cell expression matrices were processed using the R package Seurat (version 4.3.0)[9]. Normalization was performed using the “logNormalize” method, with the scale factor set to 10,000 total UMIs per cell. Quality control filtering was applied to exclude cells with fewer than 1,000 reads, fewer than 200 genes, more than 15% mitochondrial reads, or fewer than 3 cells. The “DoubletFinder” package was employed to predict doublets within the dataset, which were subsequently filtered out. In total, 19,663 cells were taken into analysis, comprising 9,119 cells from the mother and 10,544 cells from the proband.

After normalization, the two datasets were integrated using the “RunMultiCCA” function to mitigate batch effects and achieve seamless merging. Principal Component Analysis (PCA) was conducted using the highly variable genes identified by the Seurat function “FindVariableGenes” with default parameters. UMAP transformation was performed on selected principal components using the “RunUMAP” function, with a default perplexity value of 30. To identify cell clusters, the “FindNeighbors” function was used to assess neighbor relationships based on the first 30 dimensions, followed by clustering analysis with the “FindClusters” function at a resolution of 0.6. Marker genes for each cluster were identified using the Seurat function “FindAllMarkers,” applying the Wilcoxon rank sum test with a minimum percentage threshold of 0.25, a log fold-change threshold of 0.25, and a return threshold of 0.001. Additionally, clusters of the same cell type were selected for re-UMAP analysis, graph-based clustering, and marker analysis in order to explore these populations in more detail.

**Differential gene expression and enrichment analysis**

To identify genes differentially expressed across distinct cell clusters, we utilized the “FindMarkers” function in the Seurat package. Enrichment analyses were then performed to elucidate the biological roles of these genes and their involvement in cellular pathways, using the “clusterProfiler” package with a focus on the GO and KEGG databases. The results were visualized using the ggplot2 package.

**References**

1. Li H, Durbin R. Fast and accurate long-read alignment with Burrows-Wheeler transform. Bioinformatics. 2010 Mar 1;26(5):589-95.

2. DePristo MA, Banks E, Poplin R, et al. A framework for variation discovery and genotyping using next-generation DNA sequencing data. Nat Genet. 2011 May;43(5):491-8.

3. Chen Y, Chen Y, Shi C, et al. SOAPnuke: a MapReduce acceleration-supported software for integrated quality control and preprocessing of high-throughput sequencing data. Gigascience. 2018 Jan 1;7(1):1-6.

4. Kim D, Langmead B, Salzberg SL. HISAT: a fast spliced aligner with low memory requirements. Nat Methods. 2015 Apr;12(4):357-60.

5. Langmead B, Salzberg SL. Fast gapped-read alignment with Bowtie 2. Nat Methods. 2012 Mar 4;9(4):357-9.

6. Liao Y, Smyth GK, Shi W. featureCounts: an efficient general purpose program for assigning sequence reads to genomic features. Bioinformatics. 2014 Apr 1;30(7):923-30.

7. Wang L, Feng Z, Wang X, et al. DEGseq: an R package for identifying differentially expressed genes from RNA-seq data. Bioinformatics. 2010 Jan 1;26(1):136-8.

8. Zheng GX, Lau BT, Schnall-Levin M, et al. Haplotyping germline and cancer genomes with high-throughput linked-read sequencing. Nat Biotechnol. 2016 Mar;34(3):303-11.

9. Butler A, Hoffman P, Smibert P, et al. Integrating single-cell transcriptomic data across different conditions, technologies, and species. Nat Biotechnol. 2018 Jun;36(5):411-420.
