# Supplementary material for: Identification of a splice site mutation in IL2RG in a Chinese boy with X-linked severe combined immunodeficiency
Source: Genes Dis. 2025 Jan 4;12(5):101515. doi: 10.1016/j.gendis.2025.101515 (PMC12148565; doi:10.1016/j.gendis.2025.101515)
Supplement: Multimedia component 2 [file mmc2.docx]

Supplementary Table 1: Laboratory immunological data of the patient.

| Test | Value | Normal range for child |
| --- | --- | --- |
| T Lymphocyte (×10^3^/ul) | 0.481 | 1.3-2.2 |
| T Lymphocyte (%) | 59.3 | 65-79 |
| CD4+ T Lymphocyte(×10^3^/ul) | 0.022 | 0.6-1.1 |
| CD4+ T Lymphocyte (%) | 2.7 | 34-52 |
| CD8+ T Lymphocyte(×10^3^/ul) | 0.003 | 0.5-1 |
| CD8+ T Lymphocyte (%) | 0.3 | 21-39 |
| IgA (mg/dl) | 1.6 | 14-138 |
| IgG (mg/dl) | 1308.5 | 360-1060 |
| IgM (mg/dl) | 6.2 | 38-144 |
| C3 (mg/dl) | 146.3 | 79-152 |
| C4 (mg/dl) | 38.7 | 16-38 |
