# Supplementary material for: Identification of a splice site mutation in IL2RG in a Chinese boy with X-linked severe combined immunodeficiency
Source: Genes Dis. 2025 Jan 4;12(5):101515. doi: 10.1016/j.gendis.2025.101515 (PMC12148565; doi:10.1016/j.gendis.2025.101515)
Supplement: Multimedia component 3 [file mmc3.docx]

Supplementary Table 2. Splice site mutations reported as pathogenic or likely pathogenic in ClinVar.

| Gene(s) | ID | Variant | Exon | dbSNP ID | GRCh38 Location | Classification |
| --- | --- | --- | --- | --- | --- | --- |
| IL2RG | NM_000206.3 | c.855-1G>A | 7 | rs1556329822 | 71108347 | Pathogenic |
| IL2RG | NM_000206.3 | c.855-2A>C | 7 | rs2147746826 | 71108348 | Pathogenic |
| IL2RG | NM_000206.3 | c.758-1G>C | 6 | rs886042051 | 71108696 | Pathogenic |
| IL2RG | NM_000206.3 | c.758-1G>A | 6 | rs886042051 | 71108696 | Pathogenic |
| IL2RG | NM_000206.3 | c.758-2A>G | 6 | rs2147747509 | 71108697 | Pathogenic/Likely pathogenic |
| IL2RG | NM_000206.3 | c.455-2A>C | 4 | rs2092260728 | 71110297 | Pathogenic |
| IL2RG | NM_000206.3 | c.270-1G>T | 3 | rs193922346 | 71110689 | Pathogenic/Likely pathogenic |
| IL2RG | NM_000206.3 | c.116-1G>A | 2 | rs2147751144 | 71111051 | Likely pathogenic |
| IL2RG | NM_000206.3 | c.116-2A>G | 2 | rs2147751146 | 71111052 | Likely pathogenic |
| IL2RG | NM_000206.3 | c.924+1G>A | 7 | rs886041333 | 71108276 | Pathogenic |
| IL2RG | NM_000206.3 | c.854+2T>C | 6 | rs2147747293 | 71108597 | Likely pathogenic |
| IL2RG | NM_000206.3 | c.594+2_594+3del | 4 | rs1602289183 | 71110153 - 71110154 | Pathogenic |
| IL2RG | NM_000206.3 | c.594+1G>A | 4 | - | 71110155 | Pathogenic |
| IL2RG | NM_000206.3 | c.454+1G>A | 3 | rs1569480018 | 71110503 | Pathogenic |
| IL2RG | NM_000206.3 | c.269+1G>A | 2 | - | 71110896 | Pathogenic |
| IL2RG | NM_000206.3 | c.269+1G>T | 2 | rs2092262300 | 71110896 | Pathogenic/Likely pathogenic |
| IL2RG | NM_000206.3 | c.115+2T>C | 1 | rs2147751760 | 71111423 | Pathogenic |
| IL2RG | NM_000206.3 | c.115+1G>T | 1 | rs2147751762 | 71111424 | Pathogenic |
